# Parallel organization of contralateral and ipsilateral prefrontal cortical projections in the rhesus monkey

**DOI:** 10.1186/1471-2202-6-32

**Published:** 2005-05-03

**Authors:** Helen Barbas, Claus C Hilgetag, Subhash Saha, Caterina R Dermon, Joanna L Suski

**Affiliations:** 1 Department of Health Sciences, Boston University, Boston, MA, USA; 2 InternationalUniversity of Bremen, Bremen, Germany; 3 University of Crete, Iraklion, Crete, Greece

## Abstract

**Background:**

The neocortical commissures have a fundamental role in functional integration across the cerebral hemispheres. We investigated whether commissural projections in prefrontal cortices are organized according to the same or different rules as those within the same hemisphere, by quantitatively comparing density, topography, and laminar origin of contralateral and ipsilateral projections, labeled after unilateral injection of retrograde tracers in prefrontal areas.

**Results:**

Commissural projection neurons constituted less than one third of the ipsilateral. Nevertheless, projections from the two hemispheres were strongly correlated in topography and relative density. We investigated to what extent the distribution of contralateral projections depended on: (a) geographic proximity of projection areas to the area homotopic to the injection site; (b) the structural type of the linked areas, based on the number and neuronal density of their layers. Although both measures were good predictors, structural type was a comparatively stronger determinant of the relative distribution and density of projections. Ipsilateral projection neurons were distributed in the superficial (II-III) and deep (V-VI) layers, in proportions that varied across areas. In contrast, contralateral projection neurons were found mostly in the superficial layers, but still showed a gradient in their distribution within cortical layers that correlated significantly with cortical type, but not with geographic proximity to the homotopic area.

**Conclusion:**

The organization of ipsilateral and contralateral prefrontal projections is similar in topography and relative density, differing only by higher overall density and more widespread laminar origin of ipsilateral than contralateral projections. The projections on both sides are highly correlated with the structural architecture of the linked areas, and their remarkable organization is likely established by punctuated development of distinct cortical types. The preponderance of contralateral projections from layer III may be traced to the late development of the callosal system, whose function may be compromised in diseases that have their root late in ontogeny.

## Background

The primate cerebral cortex constitutes a vast communication network of ipsilateral and contralateral corticocortical connections. Although fewer in number, contralateral projection neurons, which course through the corpus callosum and the anterior commissure, have elaborate dendritic trees [1], and are critical for functional integration of the hemispheres [reviewed in [2-5]].

There is general agreement that commissural projections originate mostly from the homotopic area, and to a lesser extent from neighboring areas [e.g., [6-9]], and involve predominantly neurons in supragranular layers [reviewed in [10-12]]. It has been suggested that geographic distance is a determinant of the existence and relative laminar origin of ipsilateral corticocortical connections [13,14]. In an alternative hypothesis, the pattern of connections depends on the cortical type of the linked areas [15]. Categorical types of cortices can be determined by the number of cortical layers, thickness of layer IV, and density of neurons and other cellular markers [16]. Within this scheme, ipsilateral projections emanate from layers II-III when issued from areas with more layers, or denser layer IV, in comparison with the area of termination. In the reverse direction, projection neurons originate predominantly in layers V-VI. This hypothesis has received support in the ipsilateral connections of prefrontal areas with each other [15], and with distant sensory and association areas [17-20].

Here we tested whether geographic proximity or cortical type best explains the pattern of commissural projections linking prefrontal cortices. The prefrontal cortex is an ideal model system to investigate patterns of commissural projections because it has distinct lateral, orbitofrontal and medial sectors, which vary by distance, structural type, and pattern of ipsilateral interconnections [15]. For example, 'limbic' areas in posterior orbitofrontal and medial prefrontal regions have fewer layers and lower cell density than lateral prefrontal areas, which are eulaminate [16]. Accordingly, when limbic areas issue ipsilateral projections to eulaminate areas they do so overwhelmingly from layers V-VI [e.g. [15]]. In the reverse direction projections originate mostly in layers II-III [15].

Previous studies on patterns of commissural projections relied mostly on qualitative data, and focused on lateral prefrontal areas, or sensory and association cortices, all of which are eulaminate [e.g., [21-23]; reviewed in [10,24]]. Here we exploited the robust and consistent differences in the topography and laminar origin of ipsilateral projections arising from structurally distinct prefrontal areas, and used quantitative data to address the following questions: Are neighboring areas, or areas of similar structural type, more likely to be connected across the commissures than other areas? Are the robust laminar differences interconnecting different ipsilateral prefrontal areas also reflected in the commissural projections of these cortices?

## Results

### Injection sites

**Figure 1:**
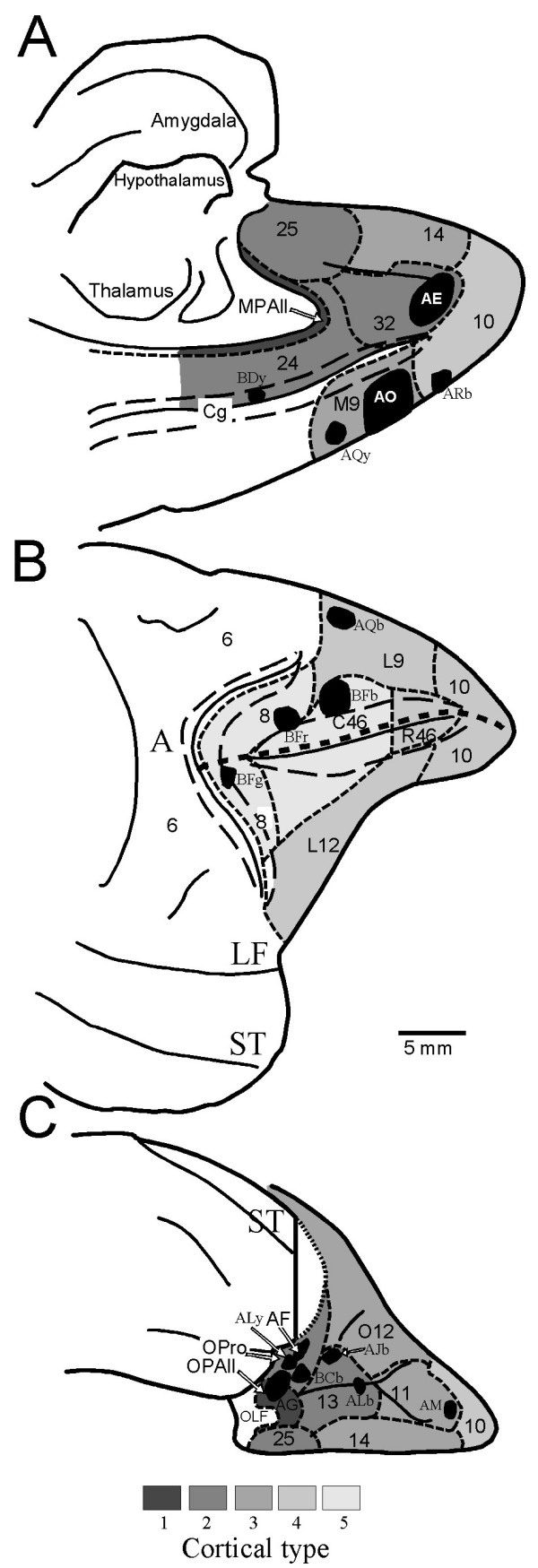
**Composite of injection sites shown on the medial (A), lateral (B) and orbital (C) surfaces of the right cerebral hemisphere**. The injection sites are superimposed on an architectonic map of the prefrontal cortex [38]. Different cortical types are depicted in shades of grey as follows: 1 (darkest grey) agranular areas with three distinguishable layers; 2, dysgranular areas with four distinguishable layers, including a poorly developed layer IV; 3–5, eulaminate areas with increasing cellular density and thickness of layer IV from levels 3 to 5. In A-C, small dashed lines demarcate architectonic areas indicated by numbers; large dashed lines depict the cortex buried in sulci. MPAll, OPAll, OPro, OLF indicate architectonic areas. Letters before architectonic areas designated by letters or numbers denote: C, caudal; L, lateral; M, medial; O, orbital; R, rostral. Other letter combinations refer to cases. Abbreviations: A, arcuate sulcus; Cg, cingulate sulcus; LF, lateral fissure; ST, superior temporal sulcus.

Evidence was provided from 12 cases, and a total of 16 prefrontal sites, which included cases with single injections of WGA-HRP and cases with injections of several distinct fluorescent dyes placed in orbital (n = 7), medial (n = 5), and lateral (n = 4) prefrontal areas, as depicted in a composite diagram of the surfaces of the prefrontal cortex in Figure 1. Further information about the injection sites is included in Additional file 1.

### Comparison of the global topography of ipsilateral and contralateral projections

**Figure 2:**
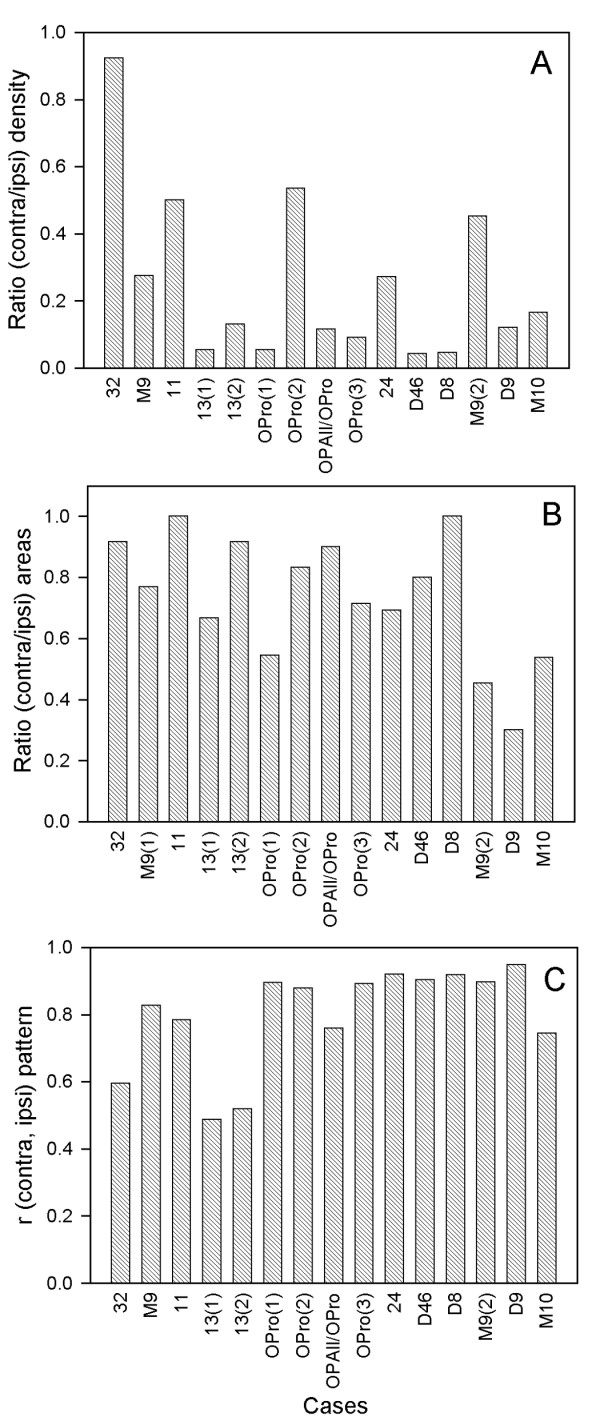
**Comparison of contralateral to ipsilateral projections**. Cases on the abscissa are identified by the area of the injection of neural tracer. Multiple cases with injection of tracers in the same area are identified by the number in parenthesis after each case. ***(A) ***Ratios of contralateral to ipsilateral projection densities. Values were calculated as the total number of contralateral neurons retrogradely labeled by an injection, divided by all labeled neurons ipsilaterally. ***(B) ***Ratios of contralateral to ipsilateral projection frequencies. Values were determined as the total number of areas with projection neurons on the contralateral side, divided by all areas with projection neurons in the ipsilateral hemisphere. Projections were included independently of their density and matching origins in the two hemispheres. ***(C) ***Strength of correlation between contralateral and ipsilateral projection patterns.

The overall number of projection neurons on the contralateral side was considerably lower than on the ipsilateral side (Fig. 2A), accounting for just 28% of ipsilateral labeled neurons (n_contra _= 24,392; n_ipsi _= 87,121). Single injections of tracer at each site labeled many cortical neurons in several ipsilateral areas (range 4–14 areas, median 12), as well as neurons in many of the same areas on the contralateral side (3–11 areas, median 9; Fig. 2B). In addition to this wide distribution of contralateral labeling, there were strong and significant correlations between the relative density patterns of ipsilateral and contralateral projections for all injection sites (Pearson's correlation, p < 0.05), except for injections in area 13 [13(1), 13 (2); cases AJb, ALb] that resulted in slightly higher probability values (p = 0.071, p = 0.051, respectively; Fig. 2C). This demonstrated that the areal distribution and density pattern of contralateral projections overlapped with a substantial subset of projections in the same areas on the ipsilateral hemisphere, despite the much lower absolute density of contralateral projection neurons. Detailed data showing the normalized density of projection neurons in different prefrontal areas, as well as the total number of projection neurons for each injection site are included in Table 1 (Additional file 2).

**Figure 3:**
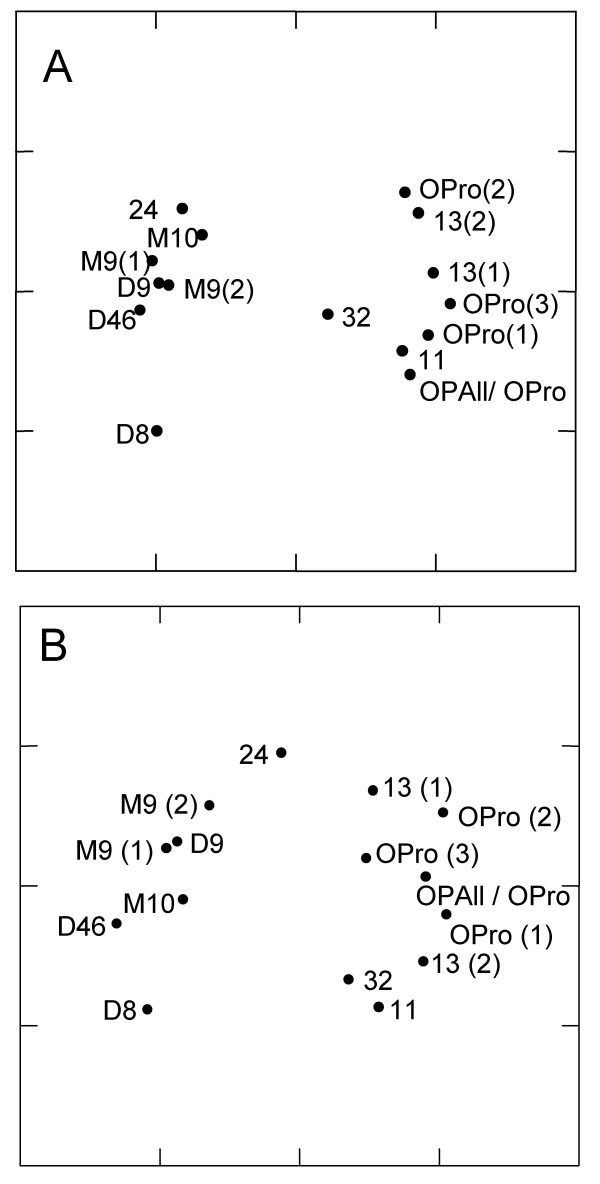
**Non-metric multidimensional scaling (NMDS), assessing the pairwise similarities of projection patterns among cases**. Cases are identified by the area of tracer injection. Multiple cases with injection of tracer in the same area are identified by the number in parenthesis after each case. Since NMDS configurations are invariant to rotation, the coordinate axes provide a scale of relative similarity, but do not prescribe specific dimensions, and are left unlabeled. ***(A) ***ipsilateral cases; ***(B) ***contralateral cases. Similarity was defined as the correlation of relative retrograde projection patterns resulting from the injections. The two main projection systems apparent in the diagram consist of predominantly mediodorsal areas (to the left), and basoventral areas (to the right) for both ipsilateral (A) and contralateral (B) projections.

The general similarity of ipsilateral and contralateral projection patterns was confirmed by an NMDS analysis, arranging injection sites by the relative similarity of their afferent connection patterns (Fig. 3). The depicted fit of areal similarities in two dimensions was high, representing 97% of the variance for the ipsilateral patterns (alienation measure: 0.10), and 83% of the variance for contralateral patterns (alienation: 0.19). On the basis of ipsilateral projection origins, prefrontal cortices were separated into two principal clusters, of mainly mediodorsal areas on the left, and mainly basoventral areas on the right-hand side (Fig. 3A). Area 32 is situated in an intermediate position between mediodorsal and basoventral areas, and similarly occupied an intermediate position, particularly for ipsilateral projections, suggesting similarity with both mediodorsal and basoventral cortices. Within the main groups, the afferent projection patterns of areas varied systematically. Specifically, changes in projection patterns appeared to follow a parallel one-dimensional gradient in both groups. The patterns of contralateral projection origins showed a very similar picture, suggesting that the general organization of ipsilateral and contralateral projections follows similar principles (Fig. 3B).

### Comparison of the regional and areal topography of ipsilateral and contralateral projections

When cortices were grouped by region, several trends emerged. Medial areas received the most widespread projections on the ipsilateral side (average = 12.4 areas), followed by orbitofrontal (average = 11.7 areas), and lateral prefrontal cortices with comparatively more restricted projections (average = 6.3 areas). The more widespread ipsilateral projections of posterior medial prefrontal and orbitofrontal 'limbic' areas, in comparison with lateral prefrontal areas, are consistent with previous findings [reviewed in [25]]. The differences noted on the ipsilateral side were also reflected in the number of areas with at least some labeled neurons on the contralateral side, with orbitofrontal areas leading (average of 9.3 areas), medial following (average 8.4 areas), and lateral cortices trailing (average 3.7 areas). The correlation in the areal pattern of projections between ipsilateral and contralateral areas did not reveal substantial regional variations. This finding reflects the overall high correlation in the areal pattern of ipsilateral and contralateral projections, described above.

**Figure 4:**
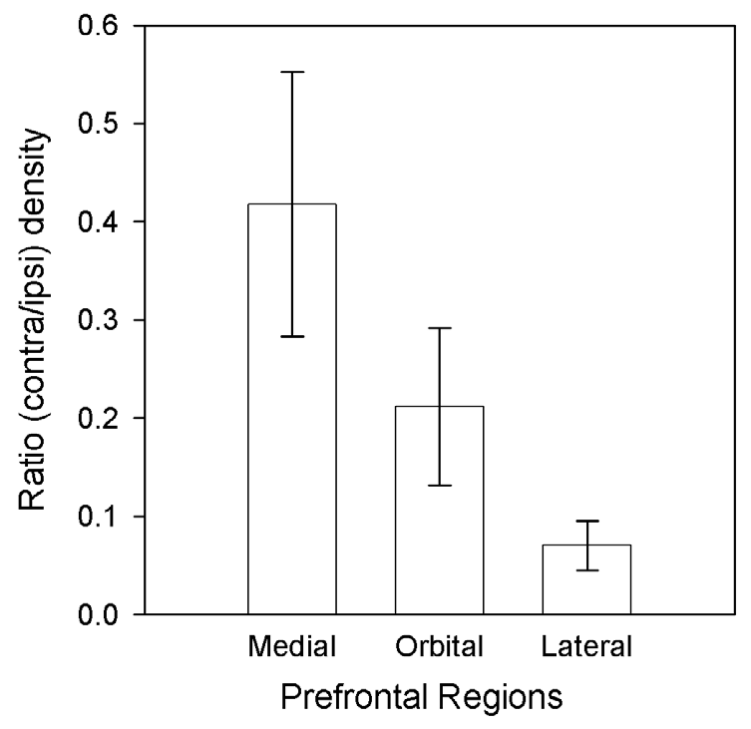
**Regional average for ratios of contralateral to ipsilateral projection densities**. The apparent trend of progressively smaller contralateral projection densities in medial prefrontal, orbital and lateral prefrontal cortices was significant (rank correlation ρ, p < 0.01).

**Figure 5:**
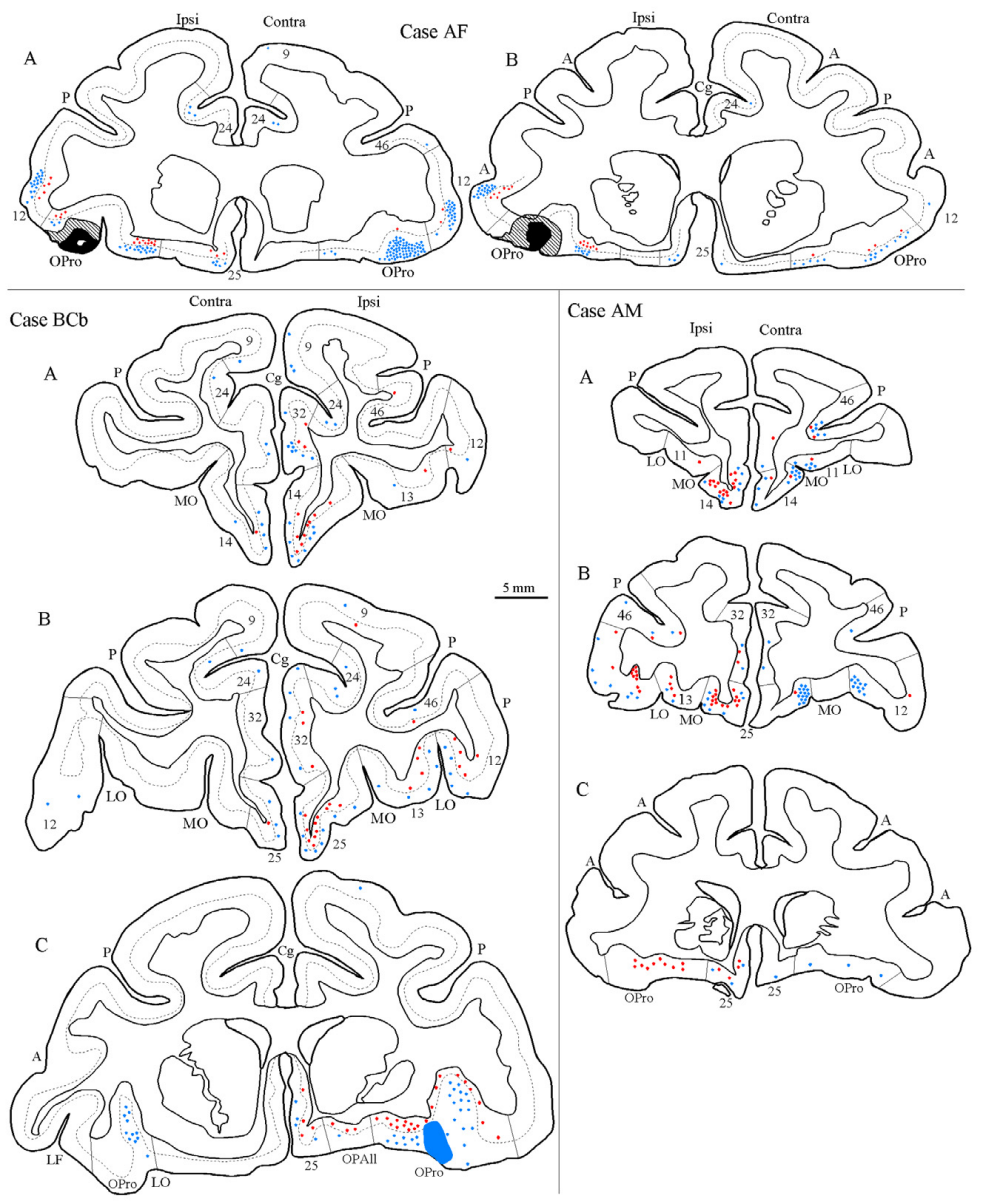
**Bilateral distribution of projections neurons directed to orbitofrontal cortices**. Coronal sections through the prefrontal cortex showing labeled neurons in ipsilateral (ipsi) and contralateral (contra) hemispheres after infection of tracers in three cases. Rostral to caudal sections are shown from left to right (case AF), and top to bottom (cases BCb and AM). ***(Top) ***The injection of HRP was in area OPro (A, B, black area) and labeled neurons are seen in layers II-III (blue dots) and layers V and VI (red dots); ***(Left) ***Injection of fast blue was in area OPro (C), blue area). ***(Right) ***Injection of HRP was in the rostral part of area 11 (not shown). Dotted lines through the cortex in the coronal sections show the bottom of layer IV. Architectonic areas indicated by letters include: OPAll, orbital periallocortex; OPro, orbital proisocortex. Other architectonic areas are indicated by numbers. Abbreviations: A, arcuate sulcus; Cg, cingulate sulcus; LF, lateral fissure; LO, lateral orbital sulcus; MO, medial orbital sulcus; P, principal sulcus.

**Figure 6:**
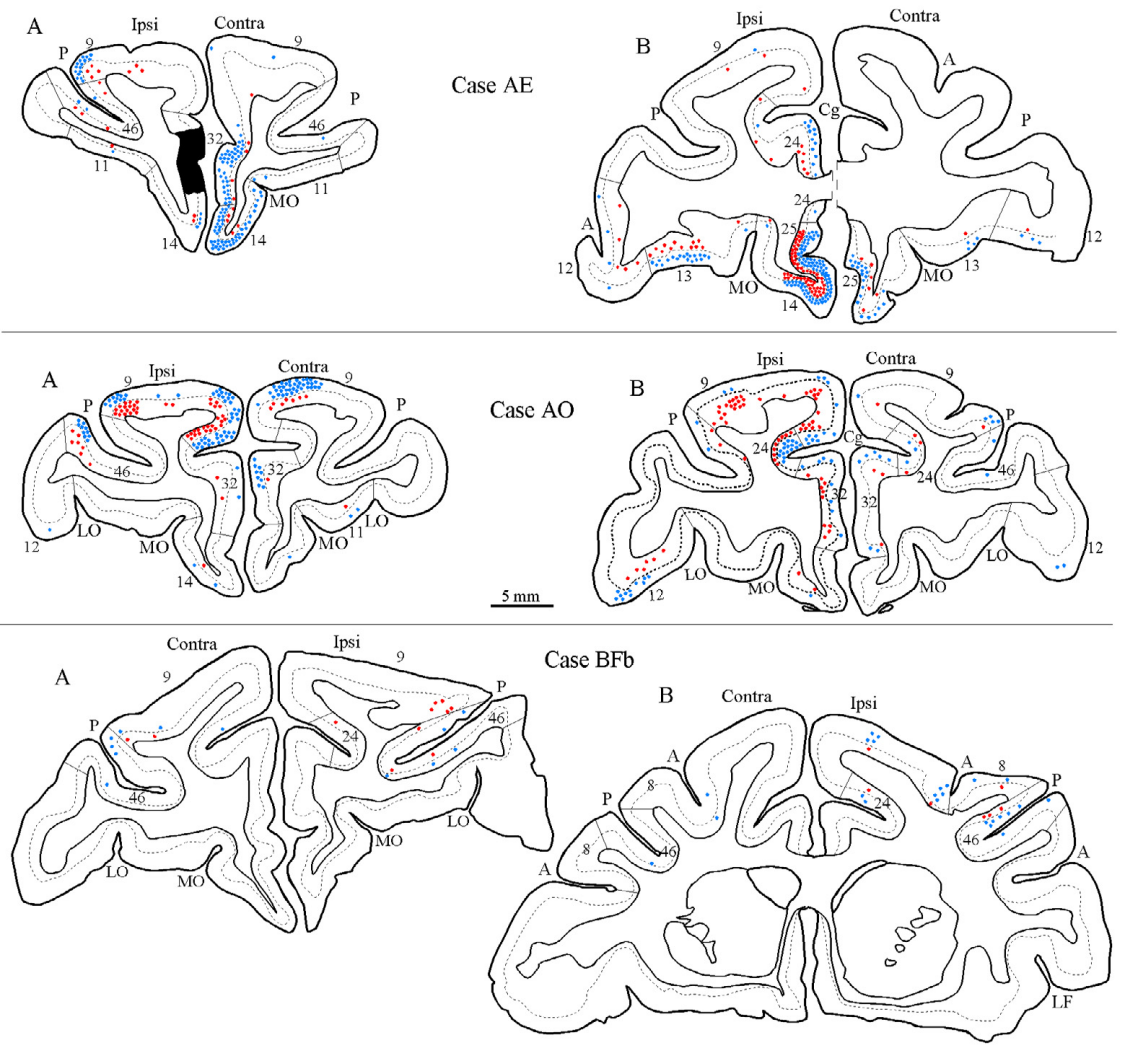
**Bilateral distribution of projection neurons directed to medial prefrontal and lateral prefrontal cortices**. Coronal sections through the prefrontal cortex showing labeled neurons in ipsilateral (ipsi) and contralateral (contra) hemispheres after infection of tracers in three cases. Labeled neurons were found in layers II-III (blue dots) and in layers V-VI (red dots). In all cases rostral sections are shown on the left, and caudal on the right. ***(Top) ***Injection of HRP-WGA (A, black area) was in medial area 32. ***(Center) ***Injection of HRP-WGA was in medial area 9 (not shown). ***(Bottom) ***Injection of fast blue was in dorsal area 46 (not shown). Dotted lines through the cortex in the coronal sections show the bottom of layer IV. Abbreviations as in Figure 5.

In contrast to the generally high correlation in the topography of bilateral projections, there was considerable variation in the ratio of the absolute density of contralateral to ipsilateral labeled neurons among injection sites (Fig. 4). Medial prefrontal sites as a group had the highest ratio (average 0.42), followed by the orbitofrontal (average 0.20), and lateral sites (average 0.07), indicating regional trends (rank correlation ρ = -0.66, p < 0.01). Figure 5 shows examples of the distribution of ipsilateral and contralateral projection neurons in prefrontal areas in cases with orbitofrontal injections (areas OPro, cases AF, BCb; area 11, case AM), and Figure 6 in cases with medial (area 32, case AE; area M9, case AO), and lateral injections (area D46, case BFb). Details of the relative density of projection neurons in different prefrontal areas for each injection site are presented in Table 1 (Additional file 2).

### Pattern in the topography of contralateral projections

We used two approaches to investigate whether there was a systematic pattern in the topography of contralateral projections. First, we investigated if contralateral projections were most prevalent with the homotopic area of the injection site in the opposite hemisphere, as well as with the corresponding neighboring areas. It has been suggested that neighboring areas are connected at a higher frequency than distant areas in the ipsilateral hemisphere [13]. For the present analysis, each area with projection neurons was given a distance rating based on the number of areal borders intervening between it and the homotopic area of the injection site. Thus, for the homotopic area the distance was 0, for an area sharing a border with the homotopic area it was 1, and for more distant areas the numbers were progressively higher.

**Figure 7:**
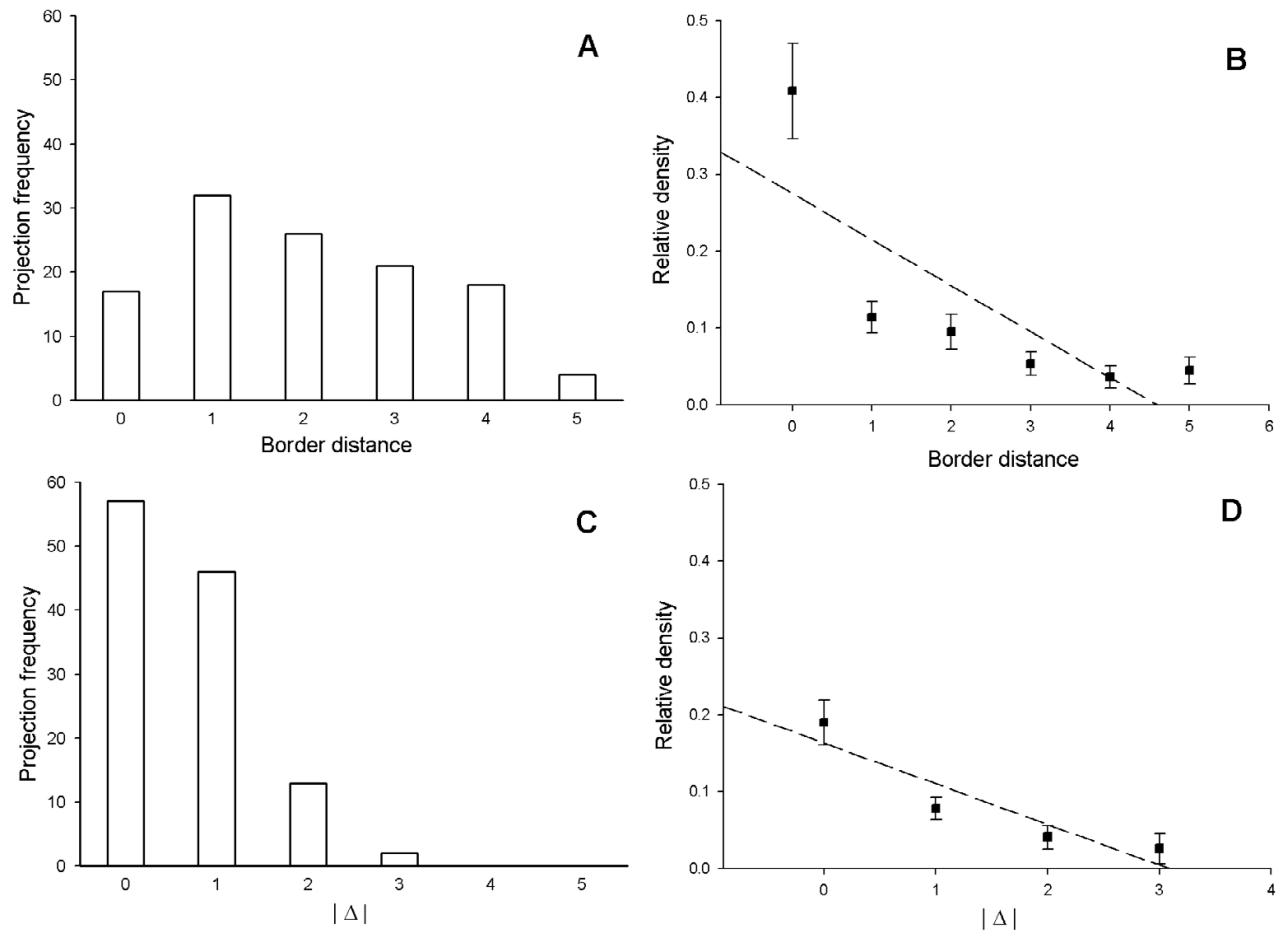
**Comparison of border distance and structural type (Δ) as determinants of the pattern of contralateral projections**. ***(A) ***Frequency of contralateral projections depending on border distance, defined as the number of borders between the contralateral area of projection origin and the area homotopic to the injection site. ***(B) ***The relationship of the density of contralateral projections to border distance (Spearman's ρ = -0.87; p (two-tailed) < 0.0002). ***(C) ***Frequency of contralateral projections as a function of the absolute structural type difference of the linked areas, delta (|Δ|). ***(D) ***The relationship of the density of contralateral projections to the type similarity between the contralateral area of origin and the ipsilateral target area, |Δ| (Spearman's ρ = -0.96; p (two-tailed) < 0.0005). Note that in panels A and C data were pooled over adjacent intervals to avoid artificial variance from sparsely filled categories. In these cases, a distance or Δ of '2', for instance, included data for distance or Δ for 2 proper as well as for 2.5.

Most contralateral projection neurons were found in the homotopic areas (border distance = 0) and their immediate neighbors (border distance = 1). Since the number of immediate neighbors was larger than that of the homotopic areas (just one for most cases), the frequency was shifted towards the next-neighbor origins, and projection neurons originated with decreasing frequency from contralateral cortices that were further away (Fig. 7A). A similar pattern was seen when the relative frequency of projections was considered (data not shown). Moreover, in terms of relative projection density, the densest contralateral projections also emanated from the immediate neighbors of the homotopic areas, showing a strong negative correlation between areal border distance and density of contralateral projections (Spearman's ρ = -0.87; p (two-tailed) < 0.0002; Fig. 7B). In all cases, the contralateral homotopic area included a significant number of projection neurons, and in most these included the densest contralateral projections (area OPro, cases BCb, ALy, AF, AG; area 13, case ALb; area 10, case ARb; medial area 9, cases AO, AQy; dorsal area 46, case BFb). In a minority of cases (cases AJb, AM, AE; Fig. 1), however, the densest projections emanated from heterotopic areas (cf. Table 1 in Additional file 2).

Heterotopic projections also originated from comparatively distant areas, including some that were separated by as many as 5 borders from the homotopic area. For example, projection neurons in substantial numbers were directed to orbitofrontal area OPro from the medially and dorsally situated areas 24, 32 and 9 (separated by 4–5 areas from the homotopic area; cases BCb, AF; Fig. 5). Similarly, relatively high numbers of projection neurons from area 24 on the medial surface were directed to orbitofrontal area 13 (separated by 3 borders; case AJb), and the orbitofrontal area OPro projected to medial area 32 (separated by 4 borders; case AE), as did orbital area 12 to medial area 9 (separated by 5 borders; case AO, Fig. 6, center).

What underlies the pattern of relatively dense projections in contralateral areas situated at a considerable distance from the homotopic area of the ipsilateral injection site? The second analysis addressed this question by considering the relationship of the structural type of pairs of connected cortices. This analysis was motivated by our previous findings that cortical type is highly correlated with the laminar pattern of ipsilateral corticocortical connections [15]. Structural type describes areas mainly by the number of identifiable layers and their relative neuronal density [16]. For this analysis the many distinct architectonic areas can be grouped into a few structural types, as shown graphically for all prefrontal areas in Figure 1. Similar to a previous study [15], we placed areas in one of five categories, the same number as the maximum number of borders between areas noted in the analysis of border distance (above). The cortical type categories ranged from 1 (agranular areas with three identifiable layers; Fig. 1, darkest grey), to 5 (eulaminate areas with six layers and the densest layer IV; Fig. 1, lightest grey). The five levels of cortical type were as follows: 1 (agranular areas OPAll and MPAll); 2 (dysgranular areas OPro, 13, 25, 24 and 32); 3 (areas 14, 11, orbital area 12, medial area 9); 4 (lateral areas 12 and 9, area 10, rostral part of area 46); and 5 (caudal part of area 46, area 8). In categories 3–5 the cortical type is eulaminate, but the density of layer IV increases from categories 3 to 5. For each pair of connected cortices we computed a value for Δ (Δ = origin level - termination level; origin was defined as the projection site, and termination as the injection site). The value of Δ for projection neurons in areas of the same type was 0 (e.g., 2-2), and so on. Type differences were treated as ordinal measures, in the same way as border distances.

The analysis showed that the great majority of projection neurons arose from areas that were of the same (|Δ| = 0) or very similar (|Δ| = 1) architectonic type as the injection site, while comparatively few projections possessed absolute Δ values greater than 2 (Fig. 7C). Moreover, the densest projections had associated Δ values of 0, and the density of all contralateral projections strongly related to the type similarity of contralateral origin and ipsilateral target area (Spearman's rank correlation, ρ = -0.96; p (two-tailed) < 0.0005; Fig. 7D). In all cases, relatively robust projections linking areas at a considerable distance (separated by >3 areal borders in the first analysis) could be explained by the similar structural relationship of the linked areas, that is, absolute type difference, |Δ|. Areas of similar type may be functionally similar as well [18], such as dorsal and ventral area 46. However, functional similarity cannot fully explain these findings, since some areas belonging to the same type have distinct functional attributes, such as anterior cingulate areas, and posterior orbitofrontal areas [reviewed in [26]].

### Comparison of the laminar origin of ipsilateral and contralateral projections

**Figure 8:**
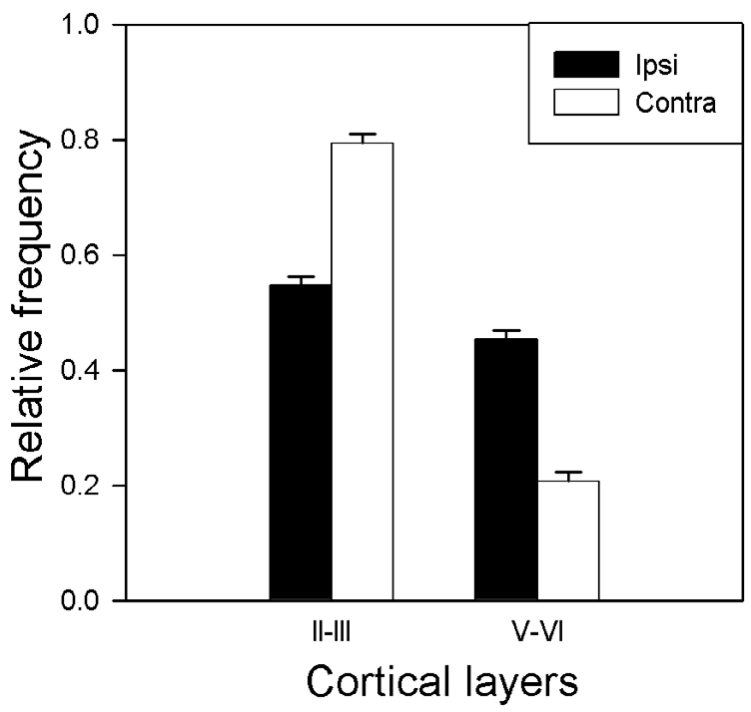
**Average proportion of laminar origins in superficial layers II-III for ipsilateral and contralateral projections**. Data from all cases were included, except for instances where a projection resulted in fewer than 20 neurons. The black and white bars, respectively, add up to 1.0.

**Figure 9:**
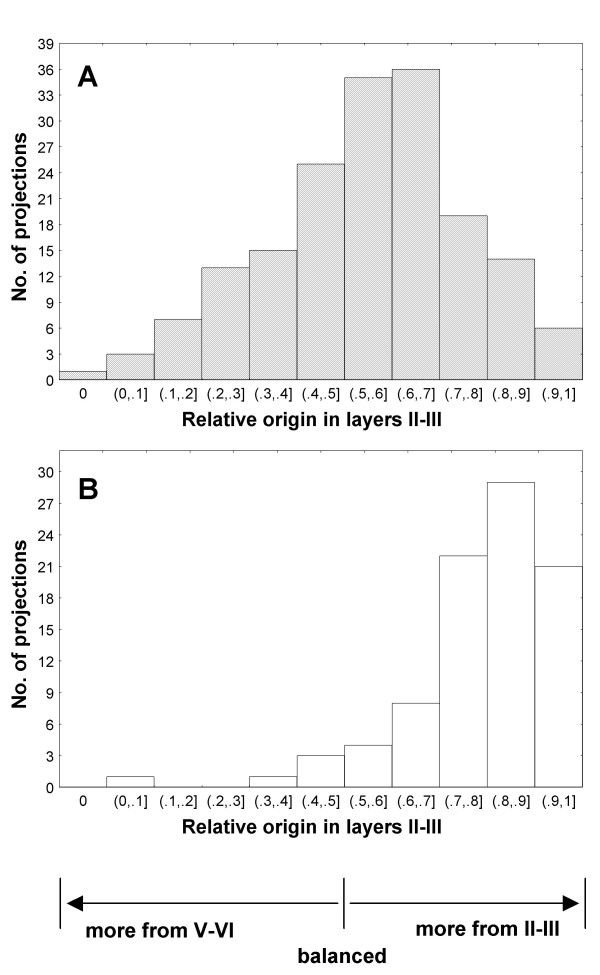
**Comparison of the relative origin of projection neurons in superficial cortical layers II-III in ipsilateral and contralateral areas**. Relative laminar origin of: ***(A) ***ipsilateral; ***(B) ***contralateral projections. The x-axis shows the proportion of projection neurons found in the upper cortical layers II-III, plotted into intervals of 10% (e.g., in the interval '.8,.9', 80%–90% of projection neurons were located in layers II-III, and the remaining 10%–20% were found in the deep layers, V-VI). Projections originating predominantly from layers V-VI are shown on the left, and projections originating in layers II-III are shown on the right. Projection neurons found in approximately equal proportions in superficial and deep layers are shown in the center. Ipsilateral projections ***(A) ***showed a broad Gaussian distribution of laminar origin patterns. By contrast, the distribution of contralateral projection origins ***(B) ***was more restricted and skewed towards the superficial layers.

**Figure 10:**
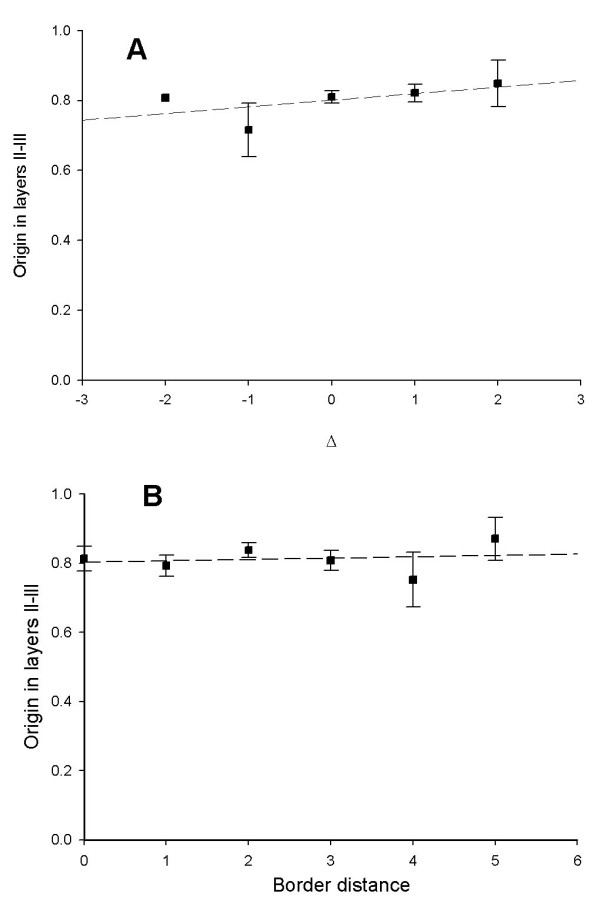
**Comparison of the relationship of laminar origin of contralateral projection neurons to structural type and border distance**. ***(A) ***The x-axis represents the type level difference, Δ), calculated as level (projection origin) – level (projection target, that is, injection site). Normalized origins of contralateral projection neurons varied significantly with Δ (Spearman's ρ, = 0.70; p < 0.04). ***(B) ***The x-axis represents the border distance from the injection site on the contralateral side (Spearman's ρ, = -0.04, p > 0.91).

**Figure 11:**
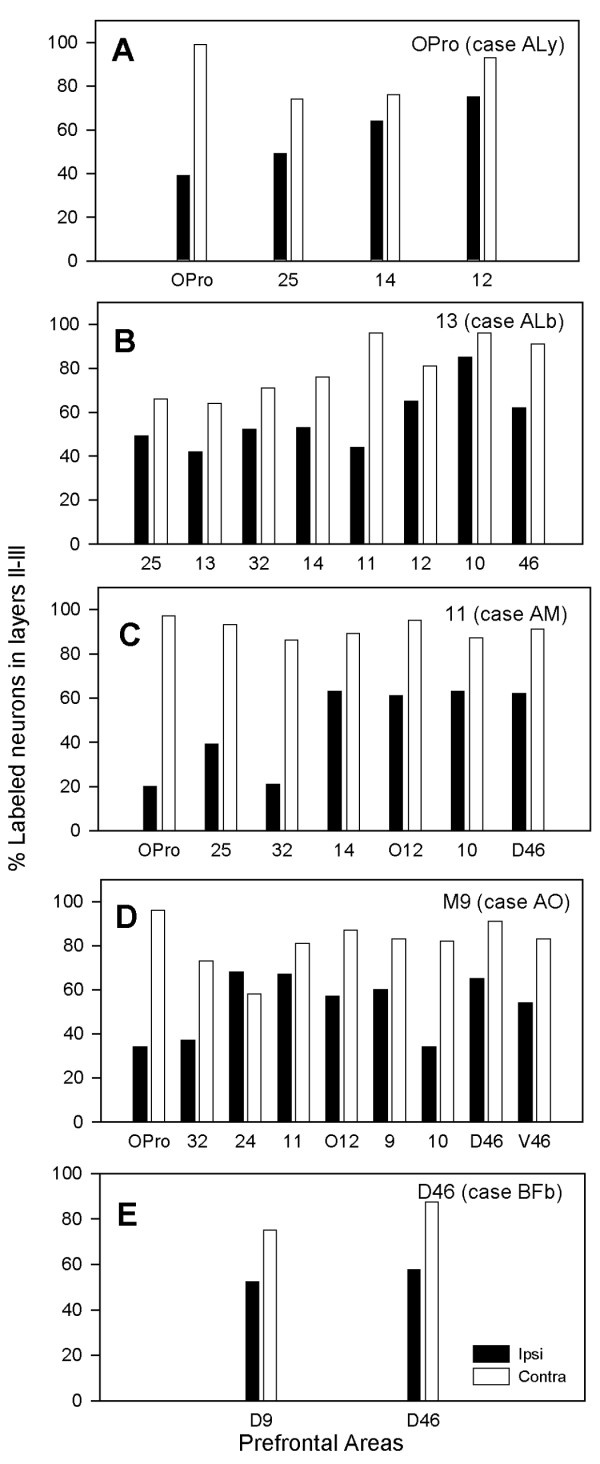
**Comparison of the distribution of projection neurons in the supragranular layers II-III in matched areas on the contralateral and ipsilateral hemispheres for individual cases**. ***(A-C) ***Areal distribution of projection neurons in cases with injection of tracer in orbitofrontal areas (area OPro, case ALy; area 13, case ALb; area 11, case AM); ***(D) ***In a case with injection of tracer in medial area 9 (case AO); ***(E) ***In a case with injection in dorsal area 46 (case BFb). In all cases the prevalence of projection neurons in layers II-III in most areas is higher on the contralateral side (silhouette bars) than on the ipsilateral side (black bars).

There were marked differences in the laminar distribution of ipsilateral and contralateral projection neurons, as shown by their average origin in superficial and deep cortical layers (Fig. 8). As already presented in earlier studies [15,17], ipsilateral projection neurons had a broad distribution based on graded laminar patterns of projections on the ipsilateral side. We previously demonstrated that the percentage of projection neurons in superficial and deep layers of ipsilateral connections varied systematically with Δ, that is, the structural type difference between origin and target area [15]. For matched contralateral areas, however, we now found a preponderance of projection neurons in the upper layers (layers III and II, with most found in layer III). Figure 9 shows the contrasted patterns of the relative frequency of projection neurons in all cases and areas in layers II-III, which resembles a Gaussian curve for the ipsilateral side (Fig. 9A), but shows a marked bias for layers II-III on the contralateral side (Fig. 9B). However, even within this restricted range of origins, the patterns were still significantly correlated with the differences in architectonic type between the area of the projection target and the area of origin, i.e., Δ (rank correlation coefficient, Spearman's ρ = 0.70; p < 0.04; Fig. 10A). Thus, the type difference between a given cortical origin and its contralateral target shaped the relative number of projection neurons arising from superficial and deep layers. A similar analysis relating laminar origin of contralateral projection neurons with geographic proximity to the homotopic area showed no correlation ρ = -0.04; p > 0.91; Fig. 10B). The comparison of the laminar distribution of ipsilateral and contralateral projections was consistent across cases, as shown in examples from individual cases (Fig. 11), and described in detail in Additional file 1 (see *Laminar distribution of projection neurons: individual cases*).

## Discussion

Patterns of contralateral projections to prefrontal cortices were highly correlated with the ipsilateral, both in topography and relative density. Although sparser in overall density than the ipsilateral, widespread commissural projections reached all prefrontal areas studied, differing regionally in density: medial prefrontal areas received the densest projections, followed by orbitofrontal, and then lateral prefrontal areas. Our findings suggest that the number of areas issuing commissural projections to prefrontal cortices surpasses the sensory areas [reviewed in [10,24], the primary motor cortex [e.g., [23,27-32]], or even neighboring premotor areas [8,22,33,34].

Global comparison of prefrontal projections suggested that the organization of ipsilateral and contralateral projections follows very similar rules. Thus, mediodorsal and basoventral prefrontal cortices segregated into two groups, both by their ipsilateral and contralateral projection patterns. Moreover, individual prefrontal areas were distinguished by their characteristic afferent projections, which were similar for ipsilateral and contralateral inputs.

Our finding that prefrontal projections group into two main clusters by their projections is consistent with previous studies on ipsilateral prefrontal projections [e.g., [35,36]]. The somewhat different appearance of the scaling and cluster diagrams in the previous and present studies is likely due to methodological differences. For example, the previous studies were based on evaluation of qualitative data from the published literature which were obtained using several different approaches [35,36]. In contrast, our analysis was based on a uniform approach by one laboratory, using quantitative data. Finally, the previous studies were based on the map of Walker [37], whereas the present study used the map of Barbas and Pandya [38], as modified from the map of Walker.

### Determinants of topography, density, and laminar origin of commissural projections

Previous studies have indicated that commissural projections of a given area are densest in the homotopic area, and, in addition, arise from several neighboring areas which also issue ipsilateral projections to the same area [discussion in [6-8]]. Our results are generally consistent with these findings, as demonstrated by a geographic neighbor analysis of projections. However, the relationship of the cortical type of linked areas (Δ) was an even better indicator for the topography, density, and laminar origin of commissural projections. It should be noted that geographic distance correlates with similarity in structural type, since areas having similar structure are frequently neighbors (Fig. 1). However, the existence and density of projections found at a considerable distance from the injection site, in either hemisphere, is consistent with the rules of the 'structural model' [15], but not a 'distance model' [14]. This result supports a broader application of the structural model for understanding organizing features of corticocortical connections [15,17,18].

There is evidence that commissural projections originate mostly from layer III in sensory [e.g., [39-43]], inferior parietal, prefrontal, and temporal cortices [e.g., [21,44]; reviewed in [24]]. The deep layers participate in the commissural system to a lesser extent, though they may have a bigger role in some premotor areas [8,45,46], and in rodents [e.g., [47,48]].

Studies based on qualitative comparisons of ipsilateral and contralateral projections showed similar laminar distributions, with minor variations in different areas or species [e.g., [49,56]]. Most previous comparisons, however, were conducted on eulaminate areas. The present study was based on a broad spectrum of prefrontal cortices which show marked differences in the laminar pattern of their ipsilateral projections. Specifically, ipsilateral projections emanate mostly from layers V-VI of caudal medial or orbitofrontal (limbic) areas, when their destination is a eulaminate cortex. In contrast, projections in the reverse direction originate mostly from layers II-III [15]. The contrasted pattern in the laminar origin of projections in limbic prefrontal cortices provided a striking example of differences across the hemispheres, demonstrating a predominant origin in layers V-VI on the ipsilateral side, but mostly from layer III on the contralateral side. On the other hand, differences in the laminar origin of projections in eulaminate prefrontal areas of the two hemispheres were subtle, consistent with previous findings [e.g., [22]].

### The pattern of commissural projections emerges in development

The preponderance of contralateral projection neurons in layer III can be traced to the late development of this system. Even though the corpus callosum emerges between embryonic days E60-65 in rhesus monkeys, at a time when only cortical layers V and VI are present [57], the establishment of callosal projections in prefrontal cortices occurs considerably later, between embryonic days E89 and E111 [58], at a time when the superficial layers are undergoing rapid growth [reviewed in [59]]. The development of this projection system late in gestation is also seen in other areas and other mammalian species [e.g. [60-63]]. Moreover, in reeler mice, where layers develop in the reverse order [64], callosal neurons are accordingly malpositioned in the deep layers, but, as in normal mice, they have the same morphology of medium-sized pyramidal neurons which develop late in ontogeny [64,65]. Further, rats irradiated late in gestation, and acallosal mice show reduction of the supragranular layers and in the size of the corpus callosum [62,66].

Notwithstanding the preponderance of commissural projection neurons in layer III, we still could detect a significant gradient of regional variation in their laminar origin, suggesting that cortical structure is a powerful determinant of the pattern of contralateral as well as ipsilateral corticocortical connections [reviewed in [67]]. This evidence raises the question of how differences in structure emerge, and by extension, how systematic patterns of connections arise.

Variation in structure among prefrontal areas is consistent with differences in the timing of their development [16]. There is evidence that cognitive abilities that rely on the orbitofrontal cortex develop earlier than those dependent on lateral prefrontal areas [68]. Importantly, in rhesus monkeys the upper layers of limbic area 24 complete their development before the same layers in area 11, which, in turn, are completed before the upper layers of area 46 [69]. These temporal differences in development explain the lower density of the upper layers in area 24, than in area 11, which, in turn, is lower than in area 46 [16]. Since projections arise as different layers are generated, a punctuated course of development of prefrontal areas could provide a mechanism for the graded laminar distribution of projections that we showed for ipsilateral connections [15] and for contralateral projections here. Moreover, axons from topographically distant prefrontal areas belonging to the same structural type share the same position within the corpus callosum, suggesting that they develop at the same time [70]. Finally, functional studies indicate that neurons participating in the tangential organization of the cortex and which develop at the same time have correlated firing patterns and likely become connected [for review [71]].

### Implications for normal function and neuropathology

Processing across the commissures may be critical for prefrontal areas, which rely on selecting relevant information and suppressing irrelevant information to guide behavior. Evidence of direct involvement of prefrontal commissural projections emerged from physiologic studies in behaving non-human primates performing cognitive tasks within the visual domain. Thus, in macaque monkeys with posterior commissurectomy that prevented the inferior temporal cortex of one hemisphere from receiving bottom-up input from the opposite visual field, single neurons in the inferior temporal cortex in the 'blind' hemisphere responded selectively to task-related stimuli [72]. Visual information relevant to the task at hand apparently followed a circuitous route from the contralateral inferior temporal cortex to the contralateral prefrontal cortex, across the corpus callosum to the opposite prefrontal cortex, and then to the inferior temporal cortex on the side of single cell recording [72,73].

Commissural projections likely have a variety of effects at the site of termination, though most commissural axons arise from excitatory pyramidal neurons [reviewed in [10]], and synapse on spines, which are enriched in pyramidal neurons [74]. The extent of contribution of inhibitory mechanisms across the commissures is not clear, though a small number of callosal axons synapse with GABAergic dendrites in rats [75,76], and a small but significant number of callosal axons originate from inhibitory interneurons [[77,78]; reviewed in [79]]. At the functional level, there is evidence of disynaptic inhibition across the corpus callosum [reviewed in [10]], as well as monosynaptic activation of basket neurons in layers III and IV [80]. Moreover, there is evidence that callosal projection neurons are modulated by dopamine [81], a neurotransmitter system affecting behavior in prefrontal cortex [e.g., [82-85]]. Interestingly, congenitally acallosal mice are hyperactive, and, unlike normal mice, have a tendency to stay off the walls, a behavior associated with a decrease in a metabolic indicator in frontal cortex [86].

The predominance of contralateral projections in layer III may be traced to the late development of the callosal system, which also has a protracted course of myelination [57,87]. Consequently, the commissural system may be affected in disorders that have their root late in development. In schizophrenia, for example, there is evidence of ineffective transmission of information across the hemispheres for cognitive tasks [reviewed in [88]]. In addition, the corpus callosum is preferentially affected with age and in Alzheimer's disease [89,90].

## Conclusion

The organization of ipsilateral and contralateral prefrontal projection systems is remarkably similar in terms of topography and relative density of projections. The two systems vary in absolute density, with contralateral projections constituting about a third of the ipsilateral. In addition, ipsilateral projections stem from layers II-III and V-VI, whereas contralateral projections have a narrow range of laminar origins, involving mostly layer III. In both systems there is a tight correlation of projection origins with the structural similarity of the linked areas. This correlation emerges even though the laminar origins of projections in the two hemispheres differ considerably. Overall, structural similarity is a better predictor of existence/absence, relative density, and laminar origin of contralateral projections than border distance of the linked areas.

The organization of ipsilateral and contralateral connections is likely established by punctuated developmental events in structurally and functionally diverse prefrontal cortices. The preponderance of commissural projections from layer III is consistent with the late development of this system, and is likely affected preferentially in diseases that have their root late in development. Contralateral projections in the prefrontal cortical system are richer in topographic origin than in sensory or motor systems. These extensive contralateral projections may be associated with the executive functions of the prefrontal cortex in the selection and synthesis of diverse signals in behavior.

## Methods

### Surgical and histological procedures

Experiments were conducted on rhesus monkeys (*Macaca mulatta*) according to the NIH guide for the Care and Use of Laboratory Animals (NIH publication 86–23, revised, 1987). The animals were anesthetized with ketamine hydrochloride (10 mg/kg, i.m.) followed either by sodium pentobarbital administered intravenously through a femoral catheter (to effect, cumulative dose approximately 30 mg/kg), or gas anesthetic (isoflurane) after intubation, until a surgical level of anesthesia was achieved. The monkey's head was firmly positioned in a holder that left the cranium unobstructed for surgical approach. A craniotomy was made and the dura retracted to expose the cortex. All injections were made with a microsyringe (Hamilton, 5 μl) mounted on a microdrive. In each animal we injected a single prefrontal site with HRP-WGA (Sigma, St. Louis, MO; 0.05–0.1 μl, 8%), or one or more prefrontal sites with distinct fluorescent tracers [(diamidino yellow, Sigma, 3% 0.5 μl; or 10% 0.25–4 μl fast blue, Sigma; or fluororuby (dextrantetramethylrhodamine, Molecular Probes); or fluoroemerald (dextran fluorescein, Molecular Probes)].

In the HRP experiments, the monkeys were given an overdose of anesthetic (sodium pentobarbital, intravenously, to effect) 40–48 hours after injection and perfused through the heart with saline followed by 2 liters of fixative (1.25% glutaraldehyde, 1% paraformaldehyde in 0.1 M phosphate buffer at pH 7.4), followed by 2 liters of cold (4°C) phosphate buffer (0.1 M, pH 7.4). The brains were then removed from the skull, photographed, and cryoprotected in glycerol phosphate buffer (10% glycerol and 2% DMSO in 0.1 M phosphate buffer at pH 7.4) for 1 day and in 20% glycerol phosphate buffer for another 2 days. The brains were then frozen in -75°C isopentane, transferred to a freezing microtome, and cut in the coronal plane at 40 μm to produce ten series. One series of sections was treated to visualize HRP [91]. The tissue was mounted, dried, and counterstained with neutral red.

In animals injected with fluorescent dyes, the survival period was 10–18 days. The animals were given an overdose of anesthetic (sodium pentobarbital, intravenously, to effect) and perfused with saline followed by 4% formalin. The brains were removed, photographed, cryoprotected in increasing concentrations of sucrose (10–30%), frozen, and cut in the coronal plane at 40 or 50 μm thickness. Architectonic areas and their borders were determined by staining with thionin, acetylcholinesterase (AChE), or myelin.

### Data analysis

Outlines of brain sections, the location of the injection site, and the areal distribution of labeled neurons, were transferred from the slides onto paper by means of a digital plotter (Hewlett Packard, 7475A) electronically coupled to the stage of the microscope and to a PC computer. In this system movements of the microscope stage are recorded as analog signals with linear potentiometers (Vernitech, Axsys, San Diego, CA) mounted on the X and Y axes of the stage of the microscope and coupled to a power supply. The analog signals are then converted to digital signals through an analog-to-digital converter (Data Translation, Marlboro, MA) in the computer. Every other section through the cortex in one series was examined and charted.

### Reconstruction of injection sites

The cortical regions containing the injection sites were reconstructed serially by using the sulci as landmarks, as described previously [92]. References to architectonic areas of the prefrontal cortex are according to a previous study [38]. Each injection extended through the depth of the cortex to include all layers.

### Statistical analysis

For topological analyses we considered, for each injection site: the number of areas with projection neurons; absolute number of labeled projection neurons; and relative projection densities. The latter measures were derived by normalizing the number of projection neurons directed to an injection site by the total number of prefrontal projection neurons in a case for each side. To determine the laminar pattern of labeled neurons, we normalized data so that the density in the upper layers (layers II-III) and the lower layers (V-VI) was expressed as a complementary percentage of the total number of labeled neurons in that area.

In order to increase statistical reliability, we applied exclusion criteria for the different analytical approaches. Projections from a given area had to consist of at least 20 neurons to be included in the laminar analyses. Since contralateral projections were generally more sparse than the ipsilateral (see Results), the laminar analyses were based on a larger number of individual ipsilateral compared to contralateral data. No general exclusion threshold was applied for other analyses. In one case (BFg), contralateral labeling did not produce sufficient data for analysis, so both ipsilateral and contralateral data were excluded from the topological investigations.

For the investigation of principles underlying connectivity, we also determined the 'border distance' between areas, that is the number of areal borders intervening between the area homotopic to the injection site and each area with projection neurons on the contralateral side. There is little information on the exact spatial trajectory of corticocortical or commissural pathways. Calculations here followed earlier approaches [13,93] and were based on the number of areal borders underneath which fibers from the injection site must have traversed to the area of labeled neurons, assuming they took the shortest path in 3D. We also calculated the architectonic type difference, delta, Δ. The latter is based on a grouping of prefrontal cortices into five structural types, defined by the structure of areas, specifically the number and neuronal density of cortical layers [15,16]. For each pair of connected cortices we computed a value for Δ (Δ = origin level - termination level), with origin defined as the projection site, and termination as the injection site. Type differences ranged from 1 (agranular areas that lack layer IV; Fig. 1, darkest grey) to 5 (eulaminate areas with the densest layer IV; Fig. 1, lightest grey), and were treated as ordinal measures, in the same way as border distances.

In instances where projections originated from two subdivisions of an area, border distance or Δ values were computed on the basis of the average of the two subdivisions (e.g., distance from lateral area 9 to dorsal area 46 = 1; distance from medial area 9 to dorsal area 46 = 2; for projections from area 9 (both divisions) to dorsal area 46, the distance was = [1+2]/2 = 1.5). This procedure affected several prefrontal areas, including the dorsal and ventral part of areas 46, 8, or 24; the orbital and lateral parts of areas 12, 14, or 25; and the medial and lateral parts of area 9. The same method was used in one case where the injection impinged on two distinct architectonic areas (areas OPAll, OPro; case AG). For graphic presentation data were pooled over adjacent intervals, so that a distance or Δ of 2, for instance, included data for distance/Δ of 2 proper as well as for 2.5.

Analyses included calculation of ratios of contralateral to ipsilateral properties, as well as correlations and non-metric multidimensional scaling (NMDS). NMDS analyses and non-parametric correlations were performed in SYSTAT V9 and SPSS V11 for Windows (SPSS Inc.), respectively. Rank correlations (using Spearman's ρ) were employed to investigate the relationship of ordinal measures, such as type differences and border distances, with metric measures of relative projection density, or with relative projection origin within the superficial cortical layers.

The NMDS analysis used to compare the global topology of ipsilateral and contralateral projections, arranges objects in two-dimensional space, based on the high-dimensional similarities among the objects [94]. The relative proximity between items in an NMDS diagram thus represents their relative similarity. In the present study, we assessed the similarity of all injection sites based on the relative ipsilateral and contralateral projection patterns that the injections uncovered. Relative density patterns were metrically correlated for all combinations of injection sites, to derive ipsilateral and contralateral matrices of pairwise injection site similarities. The two-dimensional NMDS analyses in SYSTAT used the Guttman loss function in order to represent these similarity matrices.

## Authors' contributions

HB designed the study and drafted the manuscript. HB, SS, CRD and JLS participated in mapping pathways. CCH conducted data analyses. All authors read and approved the manuscript.

## Supplementary Material

Additional File 1 Injection sites; and distribution of ipsilateral and contralateral projection neurons in prefrontal cortices in individual cases. Click here for file

Additional File 2 Table 1 Distribution density of labeled neurons in ipsilateral and contralateral prefrontal cortices. Click here for file
