# Supplementary material for: Parallel organization of contralateral and ipsilateral prefrontal cortical projections in the rhesus monkey
Source: BMC Neurosci. 2005 May 3;6:32. doi: 10.1186/1471-2202-6-32 (PMC1134662; doi:10.1186/1471-2202-6-32)
Supplement: Additional File 1 — Injection sites; and distribution of ipsilateral and contralateral projection neurons in prefrontal cortices in individual cases. [file 1471-2202-6-32-S1.pdf]

Additional files

Additional file 1 Injection sites; and distribution of ipsilateral and contralateral projection neurons in prefrontal cortices

Injection sites

Most cases described here appeared in previous studies investigating connections with the hypothalamus [1], amygdala [2,3], thalamus [4-6], hippocampal formation [7], or other cortical areas [8,9]. Previous studies on connections with other cortical areas were restricted to the ipsilateral side. Recent studies employ the same identification codes used here, and refer to the designations used in older studies [1,5,7,8]. Four new injection sites not previously presented in other studies are described briefly here. In one of these, the injection of diamidino yellow encompassed the lower bank of the cingulate sulcus, and was confined to architectonic area 24 (case BDy; Fig. 1A). The needle mark and core of the injection were confined to the cortical mantle, although the halo of the injection spread somewhat into the underlying white matter. In another animal an injection site of fast blue was above the principal sulcus at the middle rostrocaudal extent of the sulcus, within dorsal area 46 (case BFb; Fig. 1B). In the same animal, an injection of fluororuby was within the cortex situated between the caudal extent of the principal sulcus and the upper limb of the arcuate sulcus within area 8 (case BFr; Fig. 1B). Finally, an injection of fluoroemerald was in area 8 immediately posterior to the principal sulcus (case BFg; Fig. 1B). In each of these new cases the needle mark was confined to the cortical mantle.

Distribution of ipsilateral and contralateral projection neurons in prefrontal cortices

Table 1 (Additional file 2) shows the relative density of projection neurons in different prefrontal areas for each injection site, presented as a fraction of the total number (N) of projection neurons (last column). Table 1 shows entire prefrontal areas but not their subdivisions. The main findings from this analysis are presented by region below, with references to subdivisions of architectonic areas, where relevant. Sparse projections refer to fewer than 20 neurons in a given area, and are indicated with an asterisk in Table 1 (Additional file 2).

Orbitofrontal areas

Posterior orbitofrontal areas received projections from several common ipsilateral and contralateral areas, as seen in four cases with injection in area OPAll/OPro (case AG), and in area OPro (cases ALy, AF and BCb). In all cases, dense projections arose from sites near the injection site, in the homotopic area on the contralateral side (area OPro), and in area 25 on both sides (Fig. 5). Among orbitofrontal areas, the most restricted projections were noted for the most caudal orbitofrontal site, with an injection impinging on area OPAll and OPro (case AG), where large numbers of projection neurons on both sides were seen in only three areas (OPro, area 25, area 32; Table 1). Although projection neurons were noted in several other areas on both sides (areas OPAll,13, 24 and 14), they were found in substantial numbers only on the ipsilateral side. A similar distribution of projection neurons was observed for another case with an injection in an adjacent part of area OPro (case ALy), where numerous projection neurons were noted bilaterally in four areas (homotopic area OPro, and areas 25, 14 and 12; Table 1). In this case, projection neurons were found bilaterally in areas 32 and

24 as well, though they were sparse on the contralateral side. In addition, projection neurons were noted only on the ipsilateral side in areas OPAll, MPAll, area 13, areas 9 and 10, and very few in ventral area 46. Two other posterior orbitofrontal sites received comparatively widespread projections and had several common connectional features, including projection neurons bilaterally in the homotopic area OPro, and areas 25, 32, 24, 12 and 9 (cases AF and BCb; Fig. 5). They differed only by their projection from area 13, which included labeled neurons bilaterally in case AF, but only on the ipsilateral side in case BCb (Fig. 5). In the latter case projection neurons were found bilaterally in area 14, but these were less numerous on the ipsilateral side in case AF (Table 1). In both of these cases projection neurons were found in ipsilateral area OPAll, area 46, and bilaterally in area 11, which were substantial only on the ipsilateral side. Finally, projection neurons in area 10 were found in both hemispheres in case BCb, but only on the ipsilateral side in case AF.

Similar widespread and bilateral projections were directed to area 13 (cases ALb; AJb). Like the posterior orbitofrontal areas (described above), projection neurons directed to these centrally located orbitofrontal sites originated from both sides of areas 25, 13, 32, 24, 14, 12 and 10. These two sites within area 13 differed only by the preponderance of projection neurons found in area OPro and area 24, which were numerous in both hemispheres in case AJb, but in case ALb they were found in substantial numbers only on the ipsilateral side. In addition, projection neurons in case ALb were noted in area 46 and area 11 on both sides, but were seen only on the ipsilateral side in case AJb.

Similar widespread bilateral projections were issued to a more rostrally situated orbital site within area 11 (case AM). In this case, moderate to high numbers of projection neurons were noted on both sides in areas OPro, 25, 32, 14, 12, 10 and area 46 (Fig. 5, main text). The halo of the injection site covered nearly all of area 11, and projection neurons were found in area 11 on the contralateral side. In addition, in this case projection neurons were noted on the ipsilateral side in areas 13, 9, and 24, but they were only sporadic in these areas on the contralateral side.

Medial prefrontal areas

Data were obtained from five injection sites. The most posterior medial injection site was within area 24 (BDy), which received projections from a substantial number of labeled neurons from both hemispheres from area 24. In this case projection neurons were also widely distributed bilaterally in areas 32, 9, 46, 10, 13, 12, and ventral area 8, but they were very sparse on the contralateral side (<20 neurons; Table 1). In addition, labeled neurons were noted only on the ipsilateral side in areas 25 and 11. Finally, a few scattered labeled neurons were noted bilaterally in area 14, and only on the ipsilateral side in areas MPAll and OPro.

In a rostral area 32 site (case AE), we noted densely distributed projection neurons in homotopic area 32 and the adjacent area 14 on both sides. In addition, projection neurons were found on both sides of areas 25, 11, 12, 9 and 10 (Fig. 6, main text). Projection neurons were noted in areas 24 and 46 bilaterally, though they were sparse on the contralateral side. Exclusively unilateral projection neurons were noted in ipsilateral area OPAll, and in contralateral area OPro, and only a few were found in ipsilateral area 8.

In two other medial cases the injection was in rostral (case AO) and caudal (case AQy) parts of area 9. Although the injection site was in different parts of medial area 9, these cases had similar distributions of projections, but differed in overall density (case AO had denser projections than case AQy). These two area 9 cases showed common and significant bilateral projections in other parts of area 9 and in areas 24 and 32 (Fig. 6, main text). Projection neurons were also found bilaterally in area 10, and in area 46, though they were sparse on both sides in case AQy (Table 1). In the rostral medial area 9 case (case AO) labeled neurons were found bilaterally in areas OPro, 11 and orbital area 12. In addition, in case AO bilateral projection neurons were found in area 25 and the lateral part of area 12, but they were sparse on the contralateral side. In area 8 labeled neurons were noted only on the ipsilateral side in both cases, but they were sparse in case AQy.

Similarly, substantial numbers of projection neurons were directed bilaterally to medial area 10 (case ARb), including projections from other parts of area 10, as well as areas 9, ventral area 46 and area 32 (Table 1, Additional file 2). In addition, bilateral projection neurons were found in areas 24, 11, and 12, but they constituted substantial numbers only on the ipsilateral side. Strictly ipsilateral projection neurons were noted in areas 14, 25 and dorsal area 8, and very few were noted in area 13.

Lateral prefrontal areas

Data were obtained from four injection sites. In one of these, with an injection in caudal area 9, the largest proportion of projection neurons was found on both sides in homotopic area 9 (case AQb). Projection neurons were noted in area 24, though they were sparsely distributed on the contralateral side, as were projection neurons on both sides of area 10. Projection neurons were noted strictly on the ipsilateral side in areas 32, orbital areas 14 and 12, ventral area 46, and 8, and very few were seen in medial area 25, area 13, medial area 14 and lateral area 12.

A large fraction of the projection neurons directed to dorsal area 46 (BFb) was found on both sides of adjacent parts of dorsal area 46 and dorsal area 9, followed by area 8 (Fig. 6, bottom). Sparsely distributed projection neurons were found bilaterally in the medial part of area 9. In addition, projection neurons were noted strictly on the ipsilateral side in area 24, and sparsely in the lateral and orbital parts of area 12. Projection neurons noted only on the contralateral side were rare, with a few noted only in dorsal area 10.

Two other lateral sites, with injection of distinct fluorescent dyes in dorsal area 8 (BFr) and ventral area 8 (BFg, not shown) received projections from topographically similar and restricted areas. Projection neurons directed to each of these sites were found mostly in neighboring parts of area 8 on the ipsilateral side, and were sparsely distributed in the homotopic area on the contralateral side. For one of these area 8 sites (BFr), projection neurons were also found in both hemispheres in ventral and dorsal area 46, area 9 and area 24, but they were sparse on the contralateral side. Projection neurons directed to another part of area 8 (case BFg; Fig. 1B) were even more restricted to neighboring parts of area 8 ipsilaterally, and sparsely distributed in the homotopic contralateral area 8. Projection neurons were also found strictly on the ipsilateral side in dorsal area 9 and a few were scattered sparsely in dorsal and ventral area 46, lateral area 12, and dorsal area 10.

Dense projections from heterotopic areas
In three cases the densest contralateral projections were found in heterotopic areas, and interestingly all involved medial prefrontal areas. In one of these cases with an injection in area 13, heterotopic projections in area 24 were comparable to those found in homotopic area 13 (case AJb; Table 1, Additional file 2). On the other hand, in the other two cases an injection of HRP-WGA in medial area 32 (case AE), or in orbitofrontal area 11 (case AM) we saw more labeled neurons in heterotopic area 14 than in the respective homotopic areas. This may be explained by the fact that area 14 is larger than either area 32 or area 11, having both medial and orbital sectors which were considered together in this study. In addition, areas 32 and 11 share a border with area 14, and the heavy label in contralateral area 14 may reflect uptake from the fringes of the injection site in each case, or uptake by fibers of passage in the underlying white matter.

Laminar distribution of projection neurons: individual cases
The marked differences in the laminar distribution of projection neurons originating in ipsilateral and contralateral hemispheres predominated in all cases. For example, in the cases with injection in four caudal orbitofrontal sites, the higher incidence of projection neurons in layers II-III was noted in the vast majority of prefrontal areas with substantial numbers of labeled neurons. The differences were most striking for areas where projections on the ipsilateral side originated predominantly in the deep layers. For example, in ipsilateral area 25 layers II-III included only a small percentage of the projection neurons (case AF, not shown), but included a majority on the contralateral side, a pattern seen in the projection of area 25 to the other three orbitofrontal sites as well (Fig. 11A, B, main text). The same pattern was observed in two cases with injection in area 13, and in another case with injection in area 11, where the contrasting laminar pattern of ipsilateral and contralateral projections is strikingly apparent in areas OPro, 25 and 32 (Fig. 11C, main text). A notable exception to the rule was a projection from area 9 to area OPro (case AF, not shown), where the percentage of projection neurons in layers II-III was higher on the ipsilateral than on the contralateral side, and from area 32 in the same case, where the difference between the two sides was small.

In medial areas, detailed observations were made in three cases with injections in area 24 (case BDy), area 32 (case AE) and medial area 9 (case AO). In two other cases laminar information of projection neurons could not be obtained for the ipsilateral side, as they were mapped from unstained coverslipped slides (cases AQy and ARb). In all cases where the laminar distribution of projection neurons could be compared between hemispheres, marked differences were observed, with projection neurons in the upper layers predominating on the contralateral side (n=15 of 18 observations in the three cases), as shown in the example in Figure 11D (main text; case AO). In the rare exceptions where the percentage of projection neurons in layers II-III on the ipsilateral side exceeded that of the contralateral (n=3), the difference between the two sides was small (case AE, area 12, not shown; case AO, area 24, Fig. 11D, main text), and the only exception was for area 9, where the difference was larger (case AE, not shown). Interestingly, we noted the same pattern for area 9 in its projection to orbitofrontal area OPro (case AF), as described above.

In lateral areas, detailed comparisons of the laminar distribution of projection neurons were made in three cases. In the fourth case the laminar distribution of projection neurons was not available on

the ipsilateral side, as they were mapped from unstained and coverslipped slides (case AQb). The percentage of projection neurons emanating from layers II-III on the contralateral side exceeded the matched ipsilateral side (Fig. 11E, main text). This pattern showed no exceptions, even in cases where projection neurons on the contralateral side were sparsely distributed.

References

1. Rempel-Clower NL, Barbas H: **Topographic organization of connections between the hypothalamus and prefrontal cortex in the rhesus monkey.** *J Comp Neurol* 1998, **398:** 393-419.

2. Barbas H, De Olmos J: **Projections from the amygdala to basoventral and mediodorsal prefrontal regions in the rhesus monkey.** *J Comp Neurol* 1990, **301:** 1-23.

3. Ghashghaei HT, Barbas H: **Pathways for emotions: Interactions of prefrontal and anterior temporal pathways in the amygdala of the rhesus monkey.** *Neuroscience* 2002, **115:** 1261-1279.

4. Barbas H, Henion TH, Dermon CR: **Diverse thalamic projections to the prefrontal cortex in the rhesus monkey.** *J Comp Neurol* 1991, **313:** 65-94.

5. Dermon CR, Barbas H: **Contralateral thalamic projections predominantly reach transitional cortices in the rhesus monkey.** *J Comp Neurol* 1994, **344:** 508-531.

6. Xiao D, Barbas H: **Pathways for emotions and memory I. input and output zones linking the anterior thalamic nuclei with prefrontal cortices in the rhesus monkey.** *Thalamus and Related Systems* 2002, **2:** 21-32.

7. Barbas H, Blatt GJ: **Topographically specific hippocampal projections target functionally distinct prefrontal areas in the rhesus monkey.** *Hippocampus* 1995, **5:** 511-533.

8. Barbas H: **Organization of cortical afferent input to orbitofrontal areas in the rhesus monkey.** *Neuroscience* 1993, **56:** 841-864.

9. Barbas H, Ghashghaei H, Dombrowski SM, Rempel-Clower NL: **Medial prefrontal cortices are unified by common connections with superior temporal cortices and distinguished by input from memory-related areas in the rhesus monkey.** *J Comp Neurol* 1999, **410:** 343-367.
