# Supplementary material for: Parallel organization of contralateral and ipsilateral prefrontal cortical projections in the rhesus monkey
Source: BMC Neurosci. 2005 May 3;6:32. doi: 10.1186/1471-2202-6-32 (PMC1134662; doi:10.1186/1471-2202-6-32)
Supplement: Additional File 2 — Table 1 Distribution density of labeled neurons in ipsilateral and contralateral prefrontal cortices. [file 1471-2202-6-32-S2.pdf]

Additional file 2 Table 1

Distribution density of labeled neurons in prefrontal areas

Ipsilateral

CASES	Injection sites	8	46	9	10	11	12	14	13	24	25	32	OPro	OPAll	MPAll	Total N
Orbitofrontal																
AG (l)	OPAll/OPro	0	0	<0.01*	0	<0.01*	<0.01*	0.01	0.04	0.01	0.53	0.11	0.24	0.07	0	3828
ALy (r)	OPro(1)	0	<0.01*	0.02	0.05	0	0.18	0.12	0.06	0.02	0.3	0.08	0.14	0.02	0.01	7259
AF (l)	OPro(2)	0	0.01	0.03	0.01	0.05	0.22	<0.01*	0.31	0.04	0.02	0.08	0.19	0.05	0	5203
BCb (m) (r)	OPro(3)	<0.01*	0.01	0.01	0.03	0.02	0.09	0.12	0.05	0.04	0.16	0.11	0.35	0.01	<0.01*	4658
ALb (r)	13(2)	0	0.18	0.03	0.03	0.07	0.15	0.07	0.22	0.05	0.06	0.02	0.13	<0.01*	0	7444
AJb (f) (r)	13(1)	0	<0.01	0.03	0.03	<0.01	0.15	0.14	0.16	0.06	0.16	0.1	0.16	0.02	0	19419
AM (l)	11	0	0.09	0.03	0.12	<0.01*	0.12	0.13	0.03	<0.01	0.33	0.06	0.09	0	0	4381
Medial																
BDy (m) (l)	24	0.02	0.07	0.19	0.04	0.01	0.04	<0.01*	0.01	0.49	0.02	0.11	<0.01*	0	<0.01*	2442
AE (l)	32	<0.01*	0.05	0.13	0.05	0.02	0.09	0.24	0.13	0.04	0.23	0.01	0	0.01	0	11313
AO (l)	M9	<0.01	0.12	0.35	0.02	0.11	0.04	0.08	<0.01	0.19	0.01	0.06	0.01	<0.01*	0	12243
AQy (l)	M9	0.01*	<0.01*	0.68	0.02	0	0.05	<0.01*	<0.01*	0.18	<0.01*	0.05	<0.01*	0	0	1905
ARb (l)	M10	0.01	0.16	0.12	0.22	0.05	0.09	0.07	<0.01*	0.15	0.01	0.08	0.01	<0.01*	0	3212
Lateral																
AQb (l)	D9	0.04	0.05	0.57	0.01*	0	0.03	0.02	<0.01*	0.21	<0.01*	0.07	0	0	0	1213
BFb (f) (r)	D46	0.12	0.52	0.3	0	0	0.01*	0	0	0.06	0	0	0	0	0	2133
BFr (f) (r)	D8	0.45	0.44	0.06	0	0	0	0	0	0.04	0	0	0	0	0	468

Contralateral

CASES	Injection sites	8	46	9	10	11	12	14	13	24	25	32	OPro	OPAll	MPAll	Total N
Orbitofrontal																
AG (l)	OPAll/OPro	0	0	0	<0.01*	0	<0.01*	<0.01*	<0.01*	<0.01*	0.28	0.25	0.45	0.01*	0	443
ALy (r)	OPro(1)	0	0	0	0	0	0.11	0.11	0	0.03*	0.45	0.01*	0.29	0	0	394
AF (l)	OPro(2)	0	<0.01*	0.03	0	<0.01*	0.28	0.02	0.21	0.05	0.02	0.05	0.33	0	0	2784
BCb (m) (r)	OPro(3)	0	0	0.09	0.05	<0.01*	0.05	0.18	0	0.11	0.09	0.05	0.36	0	<0.01*	422
ALb (r)	13(2)	0	0.05	0.02*	0.02	0.03	0.03	0.1	0.41	0.02*	0.28	0.06	<0.01*	0	0	972
AJb (f) (r)	13(1)	0	0	0	0.05	0	0.02	0.11	0.23	0.26	0.11	0.19	0.03	0	0	1047
AM (l)	11	0	0.14	<0.01*	0.06	0.06	0.13	0.28	<0.01*	<0.01*	0.25	0.07	0.01	0	0	2192
Medial																
BDy (m) (l)	24	0.01*	0.01*	0.01*	<0.01*	0	<0.01*	<0.01*	0.01*	0.95	0	<0.01*	0	0	0	665
AE (l)	32	0	<0.01*	<0.01	0.21	<0.01	0.01	0.38	0.04	<0.01*	0.16	0.19	<0.01	0	0	10450
AO (l)	M9	0	0.09	0.47	0.16	0.05	0.05	0.01*	0	0.06	<0.01*	0.12	0.01	0	0	3369
AQy (l)	M9	0	<0.01*	0.48	<0.01*	0	0	0	0	0.33	0	0.19	0	0	0	861
ARb (l)	M10	0	0.08	0.27	0.55	0.02*	0.01*	0	0	0.02*	0	0.05	0	0	0	532
Lateral																
AQb (l)	D9	0	0	0.92	0.03*	0	0	0	0	0.05*	0	0	0	0	0	146
BFb (f) (r)	D46	0.22	0.34	0.32	0.12*	0	0	0	0	0	0	0	0	0	0	93
BFr (f) (r)	D8	0.64*	0.27*	0.05*	0	0	0	0	0	0.05*	0	0	0	0	0	22

(l), left hemisphere; (r), right hemisphere (f), female; (m), male in cases where information on gender is available
*, <20 labeled neurons
Prefrontal areas include all subdivisions as follows: 8 (dorsal and ventral); 46 (dorsal, ventral, rostral, caudal); 9 (medial, lateral); 10 (medial, lateral, ventral); 12 (orbital, lateral); 14 (orbital, medial); 24 (dorsal, ventral) 25 (orbital, medial). For references to specific subdivisions where relevant, see text.
